# Myeloid *Wls* expression is dispensable for skin wound healing and blood vessel regeneration

**DOI:** 10.3389/fendo.2022.957833

**Published:** 2022-08-22

**Authors:** Seen Ling Sim, Antje Blumenthal, Simranpreet Kaur, Kiarash Khosrotehrani

**Affiliations:** ^1^ The University of Queensland Diamantina Institute, Translational Research Institute, Brisbane, QLD, Australia; ^2^ Mater Research Institute – The University of Queensland, Translational Research Institute, Brisbane, QLD, Australia

**Keywords:** wound healing, blood vessel regeneration, tissue-resident endothelial progenitors, *Wls*, Wnt signaling, macrophages

## Abstract

Wnt signaling controls blood vessel growth, regression and patterning during embryonic and postnatal life. Macrophages are major producers of Wnt ligands and angiogenic growth factors. It regulates vascular development and specification during embryogenesis and wound healing. Macrophage dysregulation in wound healing impairs vessel regeneration and delay wound closure. During cutaneous wound healing, the endovascular progenitors (EVPs) proliferate and differentiate into mature endothelial (D) cells in response to signals produced by perivascular cells, including macrophages, governing blood vessels regeneration. However, the role of macrophage’s Wnt production on endothelial cells, especially the EVPs during wound healing is currently unknown. Here we used a cutaneous excisional wound model in mice with conditional deletion of Wnt secretion by myeloid cells (*Wls^fl/fl^LysM-Cre^+^*) to assess the kinetics of endothelial subpopulations (including EVP), myeloid infiltration, collagen deposition and wound closure. Deletion of *Wls* expression by myeloid cells did not affect wound closure and collagen deposition, indicating that myeloid *Wls* expression does not promote wound healing and regeneration. Myeloid-specific *Wls* deletion elevated the EVP population during the peak of angiogenesis, yet without affecting blood vessel density. Wounds in *Wls^fl/fl^LysM-Cre^+^* animals showed unperturbed myeloid infiltration and differentiation. Overall, our data indicate that macrophage Wnt production shapes EVP kinetics without major relevance to wound healing. These findings extend the knowledge of macrophage and endothelial molecular crosstalk and position myeloid-derived Wnt production as a regulator of endovascular progenitor.

## Introduction

The vascular network is an essential ingredient of regenerative responses and undergoes significant changes during development and adult life. With the advancement in single-cell sequencing technologies combined with lineage tracing models, subpopulations of endothelial cells with distinct transcriptomic profiles have emerged (1). This includes a population of tissue-resident endovascular progenitor (EVP) that can self-renew and differentiate into mature/differentiated endothelial cells (D), forming an endothelial hierarchy (2). EVPs remain quiescence during tissue homeostasis but retain the capacity to regenerate damaged blood vessels in physiological and pathological conditions such as skin wound healing (2–4). Under the influence of pro-angiogenic factors, quiescence EVPs can proliferate and give rise to *de novo* blood vessels (2–4). However, the key signaling pathways that help regulate EVP quiescence and its re-activation during blood vessel regeneration remain incompletely understood. Recently, sequencing data of the homeostatic aorta has identified Wnt signaling as a prominent pathway in the EVPs compared to D cells (2). Upregulation of Wnt signaling in the EVP population during tissue homeostasis may reflect the important regulatory function of Wnt in maintaining EVP quiescence.

The Wnt signaling is key regulatory pathway of embryonic development and tissue homeostasis. It controls crucial aspects of stem cell function such as its quiescence, self-renewal, proliferation, differentiation, cell fate determination, and apoptosis in various cell types (5, 6), including endothelial cells (7, 8). Both β-catenin-dependent and -independent Wnt signaling promotes endothelial cell proliferation, vessel stability and integrity during embryonic development but also later in pathophysiological conditions (7, 9–11). Conversely, loss-of-function mutations in Wnt receptor (eg, Frizzled 4; Fzd4 and Fzd5), the Wnt chaperone Wntless (Wls) and the transcriptional co-activator β-catenin in endothelial cells reduce blood vessel density and loss of barrier function in the murine central nervous system and retina (7, 8, 10–13). These results clearly indicate that Wnt signaling is essential in endothelial cells by positively modulating endothelial cell growth and function. Nonetheless, with the identification of EVPs having upregulated Wnt component, the regulatory role of Wnt signaling in EVP stemness remains undefined.

Endothelial cell growth is regulated by pro-angiogenic and anti-angiogenic factors in the microenvironment termed the perivascular niche. Apart from pericytes and smooth muscle cells, recent studies have also identified a subset of macrophages (Mϕ) lining the abluminal site of blood vessels during tissue homeostasis at adult age in several organs (14–16). These perivascular macrophages (PVMϕ) are involved in immune surveillance, tissue regeneration, and maintenance of blood vessel integrity (14–16). Depleting PVMϕ increased bacterial invasion and compromised tissue regeneration after injury with reduced blood vessel density (14–16), highlighting that PVMϕ play a critical role in modulating endothelial function. Notably, macrophages are a major source of Wnt ligands (17). Macrophage-derived Wnt5a and Wnt7b help regulate endothelial cell apoptosis and blood vessel patterning and regression during postnatal retinal development (7, 18, 19).

Given that PVM ϕ are found in proximity of endothelial cells and a major producer of Wnt ligands, we postulated that macrophage paracrine Wnt signaling contributes to the EVP functions during vessel homeostasis and regeneration. To address this hypothesis, we utilized a well-established myeloid-specific *Wntless (Wls)* knockout mouse model – *Wls^fl/fl^LysM-Cre*  (20, 21) to study the role of macrophage-derived Wnt production on the endothelial hierarchy from progenitor to differentiated cells in skin homeostasis and cutaneous wounds.

## Materials and methods

### Animal ethics and murine models

All procedures in this study complied with the Australian Code of Practice for the Care and Use of Animals for Scientific Purposes and were approved by The University of Queensland (UQ) Health Sciences Ethics Committee (UQCCR/472/18/NHMRC). Male and female conditional *Wls* knockout mice (*Wls^fl/fl^ LysM-Cre^+^*) and their littermate control (*Wls^fl/fl^ LysM-Cre^neg^*) mice between 8 to 18 weeks of age were used (20, 21). Mice were age and gender matched in all experiments. Genotype of the *Wls^fl/fl^ LysM-Cre^+^* mice were confirmed by polymerase chain reaction (PCR) of the Cre-recombinase and *Wls*-floxed gene as previously described (20, 21). The primer sequences used for mouse genotyping were: Cre-Bro-F “CTTGGGCTGCCAGAATTTCTC” and Cre-Bro-R “CCCAGAAATGCCAGATTACG”.

### Cutaneous wound healing model

Mice were anesthetized by 2% isoflurane gas inhalation in 100% oxygen. Hair was removed using hair removing cream and hair clipper prior to wounding. Two 6 mm full thickness excisional wounds were created using a punch biopsy on the dorsal back skin on day 0 as previously described (3, 22). The wounds were then left open, and a macroscopic image of each wound was taken to measure the wound size on day 0, 1 and every other day until day 7. Wound closure and total surface were manually defined, with the surface area recorded at individual timepoints. Measurements for the surface area were calculated using ImageJ software, version 1.53 (National Institute of Health (NIH), Wisconsin, USA, RRID: SCR_003070). Mice were euthanized by CO_2_ asphyxiation. The 6mm wounds with 2mm surrounding skin were collected at day 1, 3, 5 and 7 for flow cytometry and immunofluorescence analysis.

### Tissue processing and digestion

Normal skin was collected from uninjured mice and minced mechanically before digested in 0.15mg/ml of Liberase™ (Roche, Basel, Switzerland, Cat# 05401020001) in Hanks’ Balanced Salt Solution (HBSS) for 2 hours at 37°C in a shaker. The wound granulation tissue was separated from the outer wound skin and minced mechanically. The granulation tissue was then digested in 1mg/ml of type I collagenase (Life Technologies - Gibco, California, USA, Cat# 17100017) and 1 mg/ml of DNaseI (Sigma, Missouri, USA, Cat# D5025-375KU) in HBSS for 30 minutes at 37°C in a shaking incubator (3). After digestion, single cell suspensions were filtered using a 70µm cell strainer and collected for flow cytometry.

### Flow cytometry

Flow cytometry was performed on granulation and skin cell suspensions as described previously (3). For endothelial cells phenotyping, 2.5 x 10^6^ cells were resuspended in CD16/CD32 hybridoma 2.4G2 supernatant and then stained with the following antibody cocktail: rat anti-VE-Cadherin-AF488 (clone BV13) (eBioscience, Massachusetts, USA, Cat# 5016730, RRID: AB_1210528), rat anti-mouse CD34-AF647 (clone RAM34) (BD Bioscience, New Jersey, USA, Cat# 560230, RRID: AB_1645200), rat anti-mouse CD31-PE-Cy7 (clone 390) (BD Bioscience, Cat# 561410, RRID: AB_10612003), rat anti-mouse Lineage cocktail-PerCP.Cy5.5 (BD Bioscience, Cat# 561317, RRID: AB_10612020), and rat anti-mouse CD45-BUV395 (clone 30-F11) (BD Bioscience, Cat# 564279, RRID: AB_2651134) for 30 minutes at 4°C. Myeloid cells phenotyping was performed as above by staining 1 x 10^6^ cells with rat anti-mouse F4/80-BV421 (clone T45-2342) (BD Bioscience, Cat# 565411, RRID: AB_2734779), rat anti-mouse CD11b-BV785 (clone M1/70) (BioLegend, California, USA, Cat# 101243, RRID: AB_2561373), rat anti-mouse Ly6G-PE (clone 1A8) (BD Bioscience, Cat# 551461, RRID: AB_394208), rat anti-mouse Ly6C-APC.Cy7 (clone AL-21) (BD Bioscience, Cat# 560596, RRID: AB_1727555), rat anti-mouse MHCII-FITC (cloneM5/114.15.2) (BioLegend, Cat# 107606, RRID: AB_313321), and rat anti-mouse CD45-BUV395 (clone 30-F11) (BD Bioscience, Cat# 564279, RRID: AB_2651134). Fluorescence minus one controls were used to determine the cut-off point between background fluorescence and positive populations. 7-amino actinomycin D (BioLegend, Cat# 420404) was added to all samples before cell acquisition on a BD LSRFortessa X20 (BD Biosciences). Data analysis was performed on singlet events and with dead cells excluded using FlowJo™ version 10 (BD Life Sciences, New Jersey, USA, RRID: SCR_008520).

### Immunofluorescence staining

Wounds were collected and immediately fixed in 4% paraformaldehyde at 4°C for 2 hours and embedded in Optimal Cutting Temperature (OCT) (Sakura Finetek, Tokyo, Japan). Frozen sections (10µm) were permeabilized in 0.1% Triton-X in 1xPBS for 10 minutes and blocked with blocking buffer (3% bovine serum albumin and 20% goat serum in 0.1% Tween/1xPBS) for 1 hour at room temperature. Primary antibodies were diluted in blocking buffer and sections incubated with primary antibodies: rat anti-CD31 (1:100) (BD Bioscience, Cat# 557355, RRID: AB_396660), rat anti-CD144 (1:100) (BD Bioscience, Cat# 555289, RRID: AB_395707), rabbit anti-keratin14 (1:400) (Abcam, Cambridge, UK, Cat# ab181595, RRID: AB_2811031), rabbit anti-β-catenin (1:100) (Abcam, Cat# ab6302, RRID: AB_305407), and rabbit anti-cleaved caspase-3 (1:100) (Cell Signalling, Massachusetts, USA, Cat# 9661S, RRID: AB_2341188) for 1 hour at room temperature or 4°C overnight. Primary antibodies were detected using goat anti-rabbit AlexaFluor 647 (1:500) (Life Technologies, Cat# A32733, RRID: AB_2633282) and goat anti-rat AlexaFluor 568 (1:500) (Life Technologies, Cat# A11077, RRID: AB_2534121). Slides were counterstained with DAPI and mounted with DAKO fluorescence mounting medium (DAKO, Denmark Cat# S302380-2). All images were acquired using the Nikon Spinning Disc confocal microscope (Nikon, Tokyo, Japan) or Olympus FV3000 confocal microscope (Olympus, Tokyo, Japan), and images were analyzed using the FV31S-SW software, version 2.3.1.163 (Olympus) and ImageJ, version 1.53 (NIH, RRID: SCR_003070).

### Histological analysis

Frozen sections (5µm) were rehydrated and stained with Hematoxylin and eosin (H&E) and picrosirius red. Brightfield and polarized images were scanned on the Olympus VS120 slide scanner Microscope (Olympus). Area of the granulation was measured on the H&E sections using the OlyVIA, version 3.1 (Olympus), and wound collagen deposition was quantified using the Visiopharm software, version 2017.2 (Visiopharm, Denmark).

### Bone marrow-derived macrophages

Bone marrow (BM) cells from femurs or tibias of the *Wls^fl/fl^ LysM-Cre^+^and Wls^fl/fl^ LysM-Cre^neg^* mice were collected and cultured in DMEM (Life Technologies - Gibco, Cat# 11960044) containing 10% FBS, 2 mM L-glutamine (Life Technologies - Gibco, Cat# 25030081), 1 mM sodium pyruvate (Life Technologies - Gibco, Cat# 11360070), 10 mM HEPES (Life Technologies - Gibco, Cat# 15630080), and 20 ng/ml M-CSF (ImmunoTools, Cloppenburg, Germany, Cat# 12344115) as described previously (21, 23). Fresh media was added every second day. After 6 days, adherent cells were detached using TrypLE express enzyme (Life Technologies - Gibco, Cat# 12604021) and cells were collected for RNA isolation.

### RNA isolation and quantitative real-time PCR

Total RNA from BM-derived macrophages were isolated using the Qiagen RNeasy Mini Kit (Qiagen, Hilden, Germany, Cat# 74106) according to the manufacturer’s protocol. First strand cDNA was synthesized using SuperScript™ III First-Strand Synthesis System (Invitrogen, Massachusetts, USA, Cat# 18080051). Quantitative real-time PCR was performed using SYBR Green PCR master mix (Applied Biosystem, Massachusetts, USA, Cat# 4309155) on the QuantStudio 7 real-time PCR system (Applied Biosystem) and analyzed by QuantStudio Real-Time PCR software, version 1.3 (Applied Biosystem). *Wls* mRNA expression was quantified using the ΔΔCT method, normalized to the housekeeping gene *Rpl5*. The primer sequences used for qPCR were: Rpl5-F “GGCGGCGAGAGGGTAAAAC”, Rpl5-R “GCACAGACGATCATATCCCCTTC”, Wls-F “CAAATCGTTGCCTTTCTGGTG”, and Wls-R “TTGTCACACTTGTTAGGTCCC” (20, 21).

### Statistical analysis

All data were analyzed using unpaired Student t-tests or two-way ANOVA on GraphPad Prism 8 (GraphPad, California, USA, RRID: SCR_002798) software. Graphs are presented as mean ± standard deviation (SD).

## Results

### Myeloid subsets of *Wls^fl/fl^ LysM-Cre^+^* skin was unaltered during homeostasis

**Figure 1:**
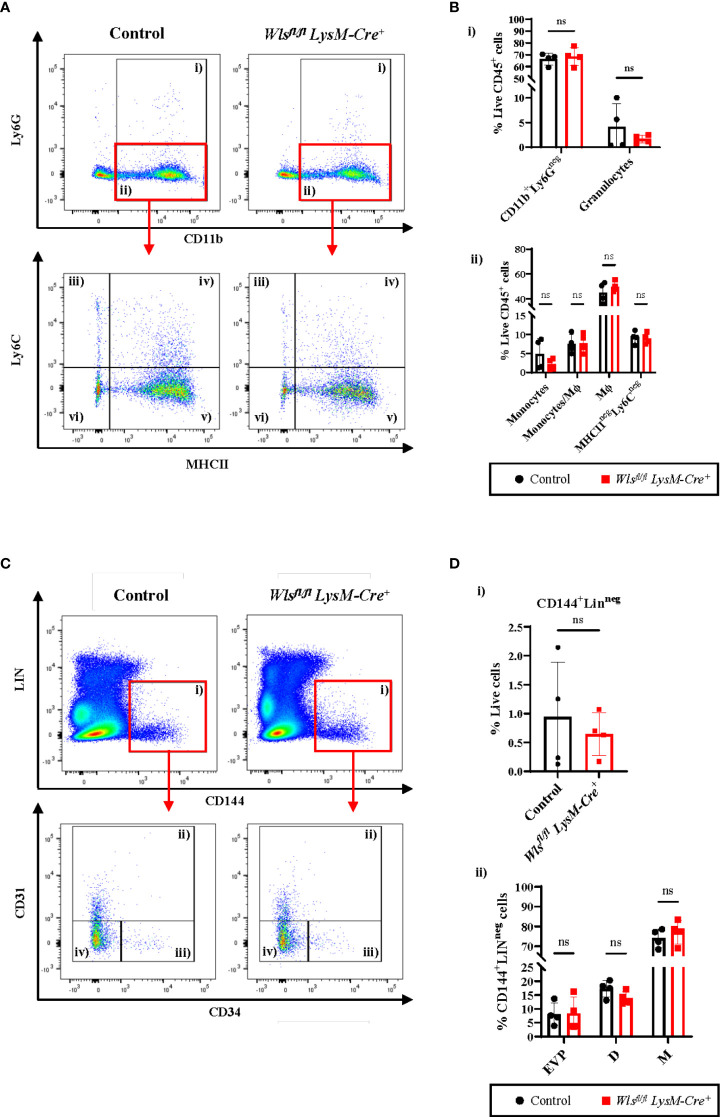
Myeloid *Wntless (Wls)* deletion does not have significant impact on the endothelial hierarchy and the myeloid populations during skin homeostasis. **(A)** Representative flow cytometry plots and gating strategy used to identify myeloid subpopulations in the skin of *Wls^fl/fl^ LysM-Cre^+^ and Wls^fl/fl^ LysM-Cre^neg^* mice. Granulocytes were gated as CD11b^+^Ly6G^+^ cells (gate i). CD11b^+^Ly6G^neg^ cells (gate ii) were subsequently gated into Ly6C^+^MHCII^neg^ monocytes (gate iii), Ly6C^+^MHCII^+^ monocytes/Mϕ (gate iv), Ly6C^neg^MHCII^+^ Mϕ (gate v) and Ly6C^neg^MHCII^neg^ cells (gate vi). **(B)** Percentage of live CD45^+^ **(i)** granulocytes and CD11b^+^Ly6G^neg^ myeloid cells consisting of **(ii)** monocytes, monocytes/Mϕ, Mϕ and Ly6C^neg^MHCII^neg^ cells of *Wls^fl/fl^LysM-Cre^+^and Wls^fl/fl^LysM-Cre^neg^* (Control) mice. **(C)** Representative flow cytometry analysis of the endothelial hierarchy in the skin of *Wls^fl/fl^ LysM-Cre^+^ and Wls^fl/fl^ LysM-Cre^neg^* mice during homeostasis. **(C)** CD144^+^Lin^neg^ cells (gate i) consists of CD31^+^CD34^+^ D (gate ii), CD31^neg^CD34^+^ EVP (gate iii) and CD31^neg^CD34^neg^ M (gate iv) cells. **(D)** Percentage of live **(i)** CD144^+^Lin^neg^ cells and **(ii)** EVPs, D and M cells in the skin of *Wls^fl/fl^LysM-Cre^+^ and the Wls^fl/fl^LysM-Cre^neg^* control littermates. No difference was observed in the total percentage of CD144^+^LIN^neg^ endothelial cells, as well as the EVP, D and M between the two groups. Data are represented as mean ± SD. Statistical analysis was performed using unpaired Student t-tests. ns= not significant. Data are representative of 4 individual animal samples.

The *Wntless (Wls)* gene encodes a chaperone protein that facilitates secretion of Wnt ligands (Wnts). To assess the role of macrophage-derived Wnt ligands on the endothelial hierarchy, we utilized the *Wls^fl/fl^LysM-Cre^+^* mouse model in which the *Wls* gene is deleted in the myeloid lineage, including macrophages. Downregulation of *Wls* mRNA expression in macrophages from the *Wls^fl/fl^ LysM-Cre+* mice compared to the *Wls^fl/fl^LysM-Cre^neg^* control littermates was confirmed **(** **Supplementary Figures 1A, B**) (20, 21). Given the importance of Wnt signaling in macrophage differentiation, polarization, and phagocytic activity (20, 21, 24, 25), we first investigated if the *Wls^fl/fl^LysM-Cre^+^* mice displayed any change in myeloid subsets of homeostatic skin. No significant difference was observed in the granulocytes [**Figures 1A(i), B(i)**] and total CD11b^+^Ly6G^neg^ cells consisting of monocytes and macrophages (Mϕ) [**Figures 1A(ii), B(i)**]. Similarly, CD11b^+^Ly6G^neg^Ly6C^+^MHCII^neg^ monocytes [**Figures 1A(iii), B(ii)**], CD11b^+^Ly6G^neg^Ly6C^+^MHCII^+^ monocytes/Mϕ [**Figures 1A(iv), B(ii)**], CD11b^+^Ly6G^neg^Ly6C^neg^MHCII^+^ macrophages [**Figures 1A(v), B(ii)**] and CD11b^+^Ly6G^neg^Ly6C^neg^MHCII^neg^ cells [**Figures 1A(vi), B(ii)**], did not show significant changes between the *Wls^fl/fl^ LysM-Cre^+^* and control mice. These data indicate that attenuating autocrine Wnt signaling does not negatively affect myeloid populations in the skin during homeostasis.

### Skin’s endothelial hierarchy remains unchanged after *Wls* deletion in myeloid cells during homeostasis

To investigate if myeloid-targeted *Wls* deletion affect blood vessel development and maintenance, we compared endothelial hierarchy in the skin between *Wls^fl/fl^ LysM-Cre^+^* and control mice during homeostasis. Flow cytometry analysis showed no difference in the total percentage of CD144^+^Lin^neg^ endothelial cells between the *Wls^fl/fl^ LysM-Cre^+^* and control littermates [**Figures 1C(i), D(i)**]. The endothelial hierarchy consisting of EVPs (endovascular progenitors), D (differentiated endothelial cells), and M (endothelial cells with mesenchymal properties) cells were identified based on CD31 and CD34 expression (3). Percentages of the CD144^+^Lin^neg^CD34^+/neg^CD31^+^ D [**Figures 1C(ii), D(ii)**], CD144^+^Lin^neg^CD34^+^CD31^neg^ EVP [(**Figures 1C(iii), D(ii)**] and CD144^+^Lin^neg^CD34^neg^CD31^neg^ M [(**Figures 1C(iv), D(ii)**] in the skin were comparable between the *Wls^fl/fl^LysM-Cre^+^* and control animals. The above results showed that macrophage Wls activity does not alter blood vessel homeostasis and development in the skin.

### Inflammatory response after wounding was not perturbed in *Wls^fl/fl^LysM-Cre^+^* mice

**Figure 2:**
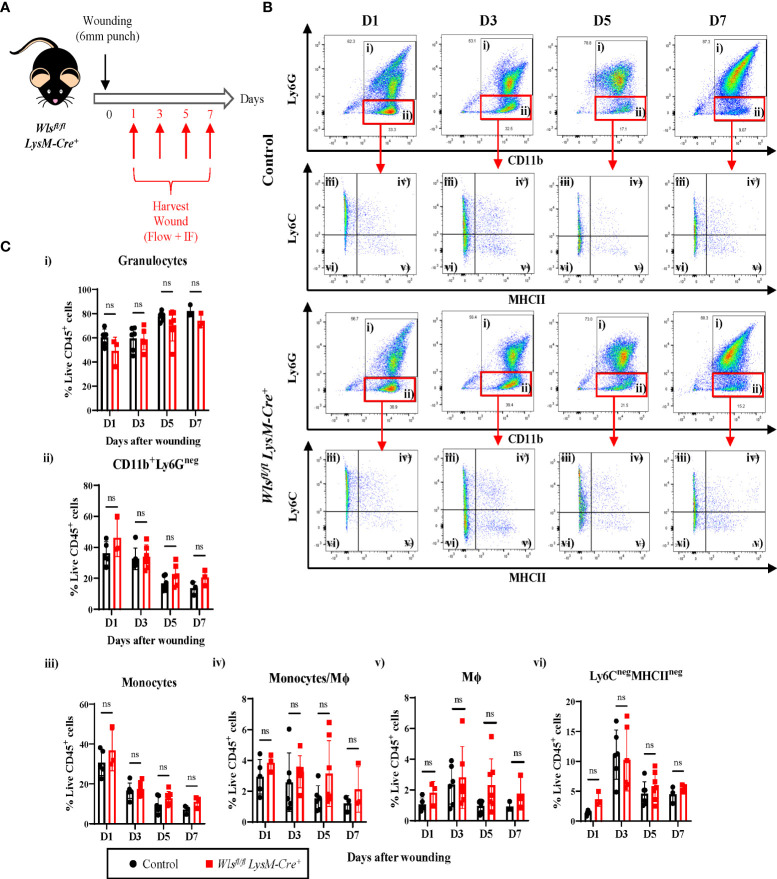
Inflammatory response in *Wls^fl/fl^ LysM-Cre^+^* mice remain unperturbed after wounding. **(A)** Schematic of the experimental timeline demonstrating the *Wls^fl/fl^LysM-Cre* wound healing time course. Two 6mm wounds were created on the murine dorsal skin using punch biopsy at day 0. Granulation tissues were collected at day 1, 3, 5, and 7 for flow cytometry and immunofluorescence analysis. **(B)** Representative flow plots showing the gating strategy used to identified granulocytes (gate i), CD11b^+^Ly6G^neg^ (gate ii) cells which are further subdivided into monocytes (gate iii), monocyte/Mϕ (gate iv), Mϕ (gate v) and Ly6C^neg^MHCII^neg^ cells (gate vi). **(C)** Flow cytometry analysis comparing granulation of *Wls^fl/fl^LysM-Cre^+^and Wls^fl/fl^LysM-Cre^neg^* mice enriched in **(i)** granulocytes and **(ii)** CD11b^+^Ly6G^neg^ myeloid cells, **(iii)** monocytes, (iv) monocytes/Mϕ, (v) Mϕ, and (vi) Ly6C^neg^MHCII^neg^ cells at all time points post wounding. Highest number of monocytes were recorded at day 1 and its percentage reduces progressively between day 3 and day 7. Monocytes/Mϕ peaked at day 3. *Wls^fl/fl^LysM-Cre^+^* has slightly higher percentage of Mϕ at day 5 (p = 0.08). Ly6C^neg^MHCII^neg^ peaked at day 3 during the inflammatory stage of the wound healing with no difference seen between the *Wls^fl/fl^LysM-Cre^+^and Wls^fl/fl^LysM-Cre^neg^* mice. Data are represented as mean ± SD. Statistical analysis was performed using unpaired Student t-tests. ns, not significant. Data are representative of 3-4 individual animal samples per time point.

While macrophage *Wls* expression is not required for myeloid and endothelial cells during homeostasis, several evidence has shown that macrophage Wnt signaling has played pivotal role in orchestrating host immune responses and tissue regeneration (24–26). Therefore, to assess the importance of myeloid-derived Wls during inflammation and tissue regeneration, we study the effect of myeloid *Wls* deletion in a cutaneous wound healing model where the inflammation and tissue regeneration process is widely characterized and well defined (27, 28). We generated two 6 mm wounds on the dorsal skin of each *Wls^fl/fl^LysM-Cre^+^* and control mouse and collect the wounds at day 1, 3, 5 and 7 (**Figure 2A**). First, we assessed the kinetics of granulocytes and monocytes/Mϕ infiltration within the granulation tissue across various phase of the wound healing process. In concordance with previous studies (29), the *Wls^fl/fl^ LysM-Cre^+^* and control littermates displayed immune cell recruitment after wounding with monocytes peaking at day 1, followed by a gradual decline between day 3 and day 7 [**Supplementary Figure 2A (iii)**]. Concurrently, the percentage of Mϕ and Ly6C^neg^MHCII^neg^ cells were highest at day 3 and decreased at day 5 [**Supplementary Figure 2A (v) and 2A (vi)**]. There were no differences between the wounds collected from *Wls^fl/fl^ LysM-Cre^+^* and control mice in the percentages of granulocytes [**Figures 2B(i), C(i)**] and total CD11b^+^Ly6G^neg^ cells [**Figures 2B(ii), C(ii)**] across all the time points. Similarly, the percentage of monocytes [**Figures 2B(iii), C(iii)**], monocytes/Mϕ [**Figures 2B(iv), C(iv)**] and Ly6C^neg^MHCII^neg^ cells [**Figures 2B(vi), C(vi)**] remained similar between the *Wls^fl/fl^ LysM-Cre^+^* and the control mice. The *Wls^fl/fl^ LysM-Cre^+^* mice showed a trend toward increased Mϕ compared to the control mice at day 5 (p=0.08) [**Figures 2B(v), C(v)**]. These results showed that myeloid-derived Wnt ligands neither impede myeloid cells infiltration nor the proportions of myeloid subpopulations after injury.

### *Wls* deficiency in myeloid cells does not impact wound healing rate

**Figure 3:**
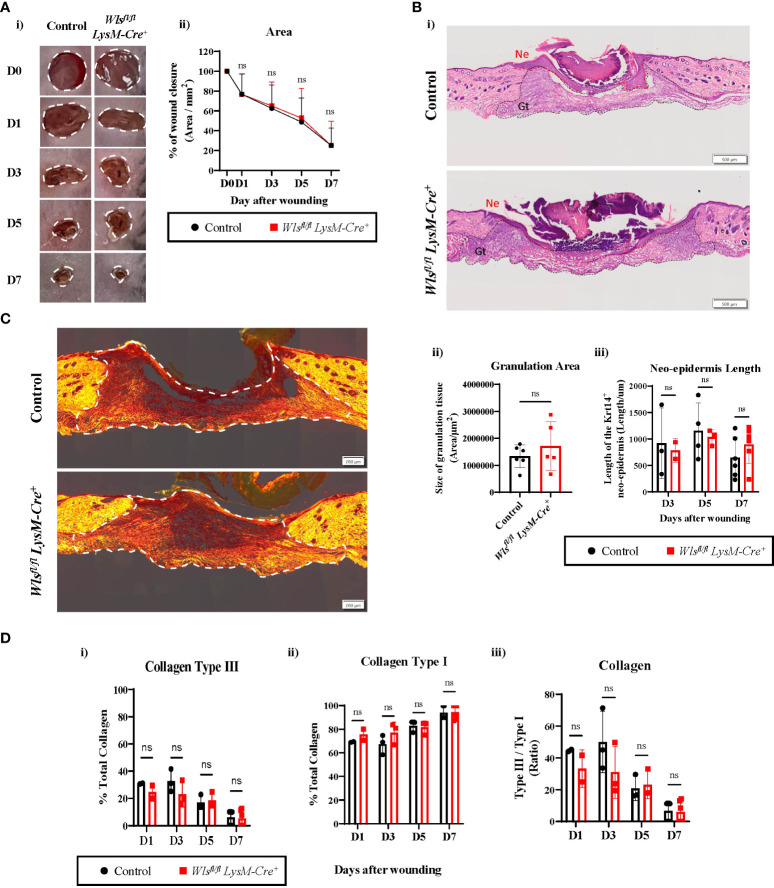
*Wls* deficiency in myeloid cells does not affect wound healing rate. **(A)(i)** Representative digital photography images showing wound closure of the *Wls^fl/fl^LysM-Cre^+^* and control mice during the seven days wound healing time course. White dotted line depicts area measured for wound closure. **(ii)** No significant difference was observed in wound closure rate between the *Wls^fl/fl^LysM-Cre^+^* and control animals at all time points (n = 4 – 23 biologically independent animals). **(B)(i)** Representative Hematoxylin and Eosin staining of skin wounds (scale bar = 500µm) demonstrating no histological difference for **(ii)** area of the granulation tissue (Gt) (n = 5-6 biologically independent animals) and **(iii)** length of the neo-epidermis (Ne) (n = 2-6 biologically independent animals) between the *Wls^fl/fl^LysM-Cre^+^* and control groups after wounding. **(C)** Representative picrosirius red staining images of day 7 wounds between control and *Wls^fl/fl^LysM-Cre^+^* animals (scale bar = 200µm). White dotted line highlights area used to measure for collagen deposition. **(D)** Collagen deposition analysis showed progressive reduction of **(i)** collagen type III to **(ii)** type I between day 1 and day 7. **(iii)** No change of collagen type III to type I ratio was observed between *Wls^fl/fl^LysM-Cre^+^* and control wound at all times (n = 2-6 biologically independent animals). Data are represented as mean ± SD. Statistical analysis was performed using unpaired Student t-tests. ns, not significant.

To determine if abrogating myeloid-derived Wnt production alters wound healing rate, we assessed the wound closure kinetics as the primary functional outcome of the wound healing process. The wounds were monitored and imaged every second day for seven days to measure the level of closure. Macroscopic image analysis of the wound area showed no differences in wound contractions at all time points between the *Wls^fl/fl^LysM-Cre^+^* and control mice [**Figures 3A(i), (ii)**]. The wound size was reduced by 20% and 80% at day 1 and day 7, respectively, in both *Wls^fl/fl^ LysM-Cre^+^* and control groups [**Figure 3A(ii)**] Likewise, size of the granulation tissue and length of the neo-epidermis that was generated from the wound edge showed no histological differences between sections of the *Wls^fl/fl^LysM-Cre^+^* and control mice wounds at all time-points during the healing process [**Figures 3B(i), (ii)** and **(iii)**]. These data suggest that the abrogation of Wnt release from myeloid cell has no direct influence on the kinetics of cutaneous wound healing.

### Myeloid-targeted *Wls* deletion did not change collagen deposition and blood vessel regeneration in cutaneous wounds

Aberrant Wnt activation has been implicated in several human fibrotic diseases, including keloid lesions and hypertrophic scars as a result of abnormal wound healing (30). To investigate if myeloid-derived Wnt ligands contributed to dermal fibrosis, we compared the collagen composition between the *Wls^fl/fl^LysM-Cre^+^and Wls^fl/fl^LysM-Cre^neg^* mice using picrosirius red staining. At the early stages of wound healing, the granulation tissue was mainly filled with collagen type III as expected [**Figures 3C, D(i)**]. As wound healing progressed from day 1 to day 7, the overall percentage of collagen type I increased with a concomitant reduction of type III collagen [**Figures 3C, D(ii)** and **(i)**]. Further histological quantification of the wounds across time showed equivalent ratios of collagen type III to type I between the *Wls^fl/fl^ LysM-Cre^+^ and Wls^fl/fl^ LysM-Cre^neg^* mice [**Figures 3C, D(iii)**]. Thus, myeloid-targeted *Wls* deletion does not change the collagen composition and wound extracellular matrix maturation during the cutaneous wound healing.

**Figure 4:**
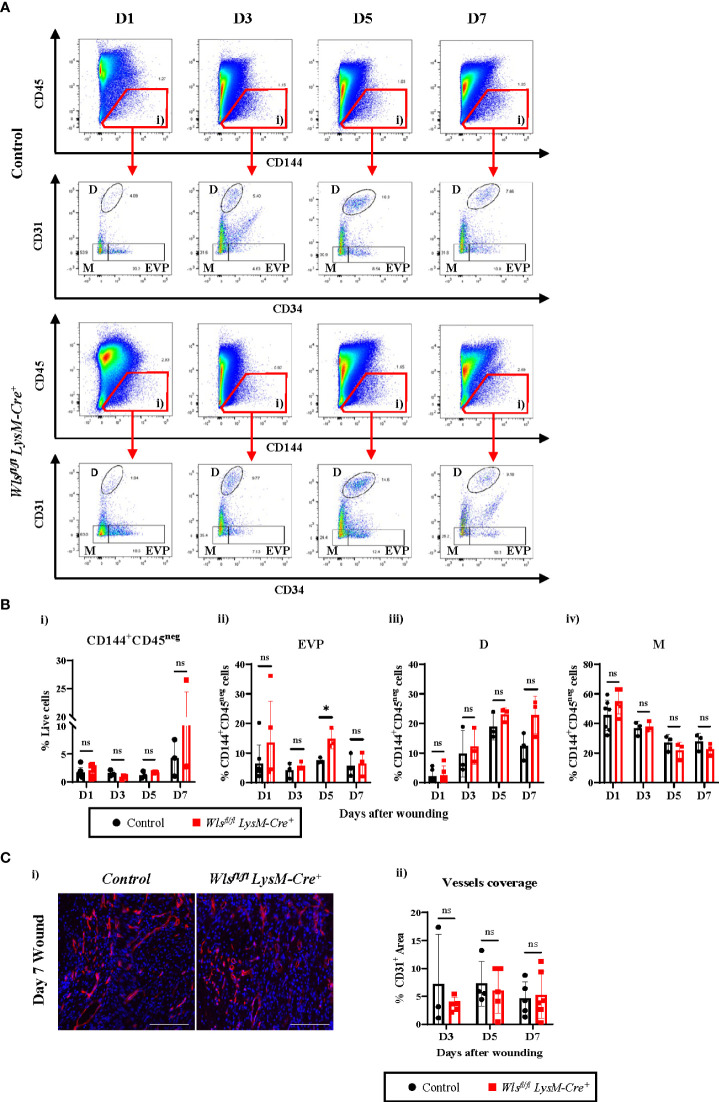
*Wls* knockout in myeloid cells does not alter blood vessel regenerations in wound healing. **(A)** Representative flow cytometry analysis of the granulation at day 1, day 3, day 5 and day 7 showing progressive changes in the endothelial hierarchy as the wound heals. Total endothelial cells were gated as CD144^+^CD45^neg^ cells (gate i). These were further fractioned as CD31^neg^CD34^+^ EVP, CD31^+^CD34^+^ D and CD31^neg^CD34^neg^ M cells. **(B)** Flow cytometry analysis showing percentage of live **(i)** CD144^+^CD45^neg^ cells and **(ii)** EVPs, **(iii)** D and **(iv)** M cells in the granulation of *Wls^fl/fl^LysM-Cre^+^* and control mice across the wound healing time course. No major alteration was observed in the percentage of the endothelial cells except EVPs which were elevated at day 5 in the *Wls^fl/fl^LysM-Cre^+^* mice. **(C)(i)** Representative immunofluorescence staining of the endothelial marker CD31 (red) and DAPI (blue) in the granulation of *Wls^fl/fl^LysM-Cre^+^ and Wls^fl/fl^LysM-Cre^neg^* mice at day 5 post wounding. (Scale bar = 100µm) **(ii)** Quantification of CD31^+^ staining in granulation at day 3, day 5 and day 7 showing no significant difference in the blood vessels of *Wls^fl/fl^LysM-Cre^+^ and Wls^fl/fl^LysM-Cre^neg^* wounds. Data are represented as mean ± SD. Statistical analysis was performed using unpaired Student t-tests. ns = not significant, * = p<0.05. Data are representative of 3-5 individual animal samples.

We next performed flow cytometry analyses to assess the endothelial hierarchy throughout the wound healing process (**Figure 4A**). Percentages of the CD144^+^CD45^neg^ population were comparable between the *Wls^fl/fl^ LysM-Cre^+^ and Wls^fl/fl^ LysM-Cre^neg^* mice with a trend towards increased numbers in the *Wls^fl/fl^LysM-Cre^+^* mice at day 7 [**Figures 4A(i), B(i)** and **Supplementary Figure 2B(i)**]. Looking further into the different endothelial subpopulations, the percentage of EVPs remained consistent between day 1 and day 7 in the control mice [**Supplementary Figure 2B(ii)**]. A higher variability was observed in EVP numbers in the *Wls^fl/fl^ LysM-Cre^+^* wounds with a notable increase at day 5 [**Supplementary Figure 2B(ii)**]. Percentage of EVP was elevated two-fold in the *Wls^fl/fl^ LysM-Cre^+^* mice compared to the control mice at day 5 [**Figures 4A, B(ii)**], and similar trends were observed on day 1 [**Figures 4A, B(ii)**]. Double staining of the apoptosis marker, cleaved Caspase-3 with CD31 on day 5 wounds showed similar number of Caspase-3^+^ endothelial cells between the control and *Wls^fl/fl^ LysM-Cre^+^* mice [**Supplementary Figures 3A, B**]. This result indicates that *Wls* deletion in myeloid cells does not affect endothelial cells and particularly EVP apoptosis. As expected, there was a significant increase in D population between day 3 and day 5, commonly considered as the peak of angiogenesis [**Figure 4B(iii)** and **Supplementary Figure 2B (iii)**]. A similar trend of increased D cells was observed in the *Wls^fl/fl^LysM-Cre^+^* and control groups [**Supplementary Figure 2B(iii)**]. We observed a reduction of D cells in the control wound at day 7 [**Figures 4A, B(iii)**], which is consistent with previous results (3). In contrast, the percentage of D cells remained high in the *Wls^fl/fl^LysM-Cre^+^* wound [**Figures 4A, B(iii)**]. This result indicates that myeloid *Wls* activity might be involved in vessels regression.

The M population reflects the endothelial to mesenchymal transition phenomenon in the wound. There was a gradual decreased in the M population across the 7 days of healing process with no significant difference identified between *Wls^fl/fl^ LysM-Cre^+^ and Wls^fl/fl^LysM-Cre^neg^* mice [**Figures 4A, B(iv**) and **Supplementary Figure 2B (iv)**]. Immunofluorescence staining of the endothelial marker, CD31, did not show any differences between *Wls^fl/fl^LysM-Cre^+^ and Wls^fl/fl^LysM-Cre^neg^* mice in CD31^+^ blood vessel coverage within the granulation site at all time points during the healing process [**Figures 4C(i), (ii)**]. Overall, the above results demonstrated that myeloid-derived Wnt ligands may impact the proportion of vascular subpopulations by modulating EVPs response to cues in the wound microenvironment, but the resulting number of blood vessels remains unchanged.

### Nuclear β-catenin remains unaltered in the endothelial cells after *Wls* knockout in macrophages

**Figure 5:**
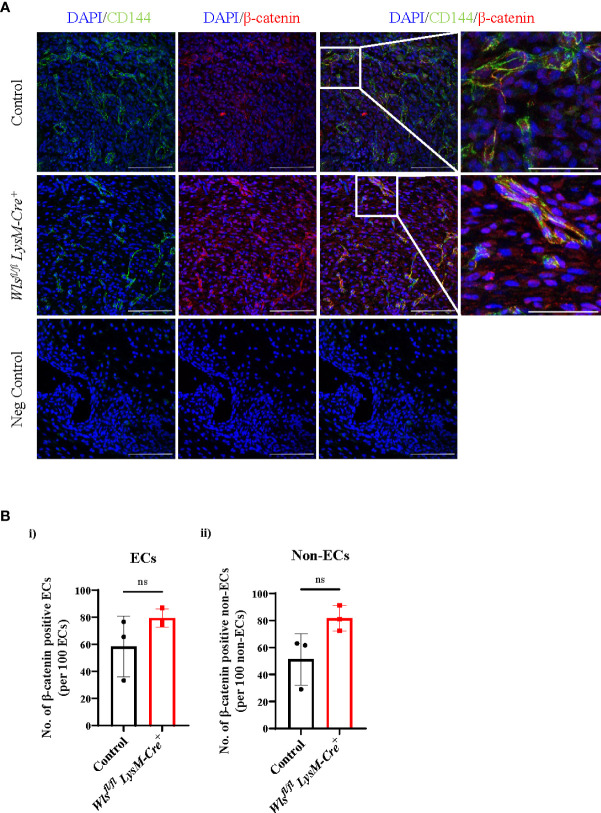
Nuclear β-catenin number in the *Wls^fl/fl^ LysM-Cre^+^* mice remain unchanged in the wound endothelial cells. **(A)** Representative immunofluorescence staining of the β-catenin (red), CD144 (green) and DAPI (blue) in the day 5 *Wls^fl/fl^LysM-Cre^+^ and Wls^fl/fl^LysM-Cre^neg^* wounds (scale bar = 100µm). White boxes highlighted CD144^+^ endothelial cells co-localized with nuclear β-catenin^+^ imaged at higher magnification (scale bar = 100µm). **(B)** Quantification of the β-catenin in the **(i)** ECs and **(ii)** non-ECs showed no difference in the number of nuclear β-catenin between the *Wls^fl/fl^LysM-Cre^+^* and control mice. Data are represented as mean ± SD. Statistical analysis was performed using unpaired Student t-tests. ns = not significant, ECs = endothelial cells. Data are representative of 3 individual animal samples.

One major axis of Wnt-mediated cellular activation is driven by β-catenin dependent Wnt signaling, which results in the nuclear translocation of the transcriptional co-activator β-catenin (31). We therefore assessed nuclear β-catenin in endothelial cells in wounds from *Wls^fl/fl^LysM-Cre^+^and Wls^fl/fl^LysM-Cre^neg^* mice. On day 5, at the peak of angiogenesis, β-catenin was activated in endothelial cells in wounds of both *Wls^fl/fl^LysM-Cre^+^ and Wls^fl/fl^LysM-Cre^neg^* mice (**Figure 5A**). Quantification of the number of nuclear β-catenin^+^ cells in endothelial and non-endothelial cells showed no significant differences between *Wls^fl/fl^LysM-Cre^+^and Wls^fl/fl^LysM-Cre^neg^* mice [**Figures 5B(i), (ii)**]. This result suggests that Wnt ligands from sources other than macrophages, such as perivascular or endothelial cells, may drive Wnt/β-catenin signaling in endothelial cells.

## Discussion

In the present study, we aimed to assess the roles of myeloid-derived Wnt ligands on endothelial hierarchy during skin homeostasis and wound healing. Our results indicate that *Wls* deletion in macrophages does not alter development of endothelial cells or the composition of myeloid cells in the normal skin at adult age. Moreover, during wound healing, despite an increase in EVP proportion, the loss of Wls activity by macrophages did not alter wound vascularization or myeloid infiltrate composition. The persistence of nuclear β-catenin in endothelial cells suggested alternative sources of Wnt ligands that drive Wnt signaling in endothelial cells.

Our results demonstrated that constitutive myeloid *Wls* deletion did not affect blood vessel development in the adult skin. *Wls^fl/fl^LysM-Cre^+^* mice had normal endothelial cell numbers in unwounded skin (**Figures 1C, D**). This observation is consistent with previous reports in which whole-mount CD31 immunofluorescence analysis of another myeloid specific *Wls* knockout model - the *Wls^fl/fl^ Csfr1-icre* embryonic skin showed no phenotypic changes in blood vessel structure and density during prenatal development (19). Extending to the existing finding, our study indicates that myeloid-derived Wnts play a negligible role in maintaining EVPs during skin homeostasis [**Figures 1C, D(ii)**]. These results suggest that myeloid-derived paracrine Wnt activity does not directly regulate CD31^+^ endothelial cell growth in the skin during development. However, we cannot exclude that other cell types or non-Wnt related ligands (e.g. Norrin) and co-receptor (TSPAN12 and Gpr124) might be involved in skin blood vessel development (10, 11, 32).

In wound healing, Wnt1, 3, 4, 5a, 10b transcripts were identified early in the full thickness wound up to day 7 (33). In our study, attenuating Wnt production from myeloid cells increased EVPs during the peak of vessel growth without affecting blood vessel density (**Figures 4A–C**). These results suggest that macrophage paracrine Wnt signal might maintain EVPs quiescence or increase EVPs apoptosis. However, the lack of difference in cleaved Caspase-3 in endothelial cells does not support an important role for apoptosis in this context (**Supplementary Figures 3A, B**). Therefore, we have shown that increased EVPs in day 5 wounds is not due to reduce endothelial cells apoptosis, and myeloid-derived Wnts is not involved in regulating endothelial cells survival. Macrophages produce localized Wnts that control blood vessel patterning and regression in specific region of the retina (18, 34). Moreover, Wnt ligands were established as key molecules of stem cell niches (eg. skin interfollicular epidermis, intestine and liver), permitting precise control of stem/progenitor cells’ self-renewal and differentiation during homeostasis and after injury (35–39). It is plausible that macrophage-derived Wnts also constitute the endothelial progenitors’ perivascular niche during wound healing and are required to control EVPs quiescence and self-renewal. Attenuation of the macrophage Wnts in the *Wls^fl/fl^LysM-Cre^+^* mice may disrupt perivascular local cues and hence, change EVPs growth kinetics as showed in our study.

Despite the increase of EVP population after myeloid *Wls* deletion, the overall blood vessel density was not affected (**Figures 4C(i), (ii)**). This may not be surprising as other cell types also express Wnt ligands after cutaneous wounding. Okuse et al. described Wnt expression in the migrating epithelial cells and dermal fibroblasts (33). Our study shows that level of nuclear β-catenin remained unaffected in the *Wls^fl/fl^LysM-Cre^+^* wound endothelial cells [**Figures 5A, B(i)**]. It is noteworthy that based on our previous RNA sequencing data, EVP and D cells also express various Wnt ligand with a more prominent expression in the EVPs. Based on this observation, we argue that Wnt ligands produced by keratinocytes, fibroblasts or endothelial cells might compensate for the loss of myeloid-derived Wnt ligands either *via* paracrine or autocrine signaling. Wnt signaling is highly complex and played vital roles in tissue regeneration and β-catenin independent or other non-Wnt related compensatory mechanisms exist to counterbalance the myeloid signaling defect. For example, apart from the macrophage Wnt7b induced ECs apoptosis (18), the endothelial RSPO3 (a Wnt signaling enhancer) was also shown to regulate vessel stability and regression in the developing retina *via* the Wnt/Ca^2+^ pathway (40). These findings have highlighted the complexity and functional redundancy of the Wnt signaling pathway exert by different cell types. Therefore, further analyses are required to elucidate the underlying molecular mechanism involved in this study.

Abrogation of Wnt signaling during wound healing delayed wound closure due to reduced epithelial proliferation, fibroblasts contraction and collagen deposition (41). In our study, myeloid *Wls* loss did not affect wound closure rate [**Figures 3A(i), (ii)**]. It is known that human cutaneous wound healed by re-epithelisation and murine wound mainly healed *via* contraction and re-epithelisation. Recent wound healing studies have suggested the use of splint in cutaneous wound healing model to prevent the panniculus carnosus contraction and can better recapitulate human wound healing. To note, our study has not adopted the splinted cutaneous wound model and the resulting effect on wound closure might involved wound contraction and re-epitheliastion. Despite the fact, length of the newly regenerated epidermis were similar between the control and *Wls^fl/fl^ LysM-Cre^+^* animals [**Figures 3B(i), (iii)**] suggesting myeloid *Wls* activity is dispensable for neo-epidermis regeneration. While re-epithelization of the wound is governed by proliferation and differentiation of epidermal cells, including progenitors that reside within the hair follicle and basal layer (42–45), Wang et al. demonstrated that β-catenin in the Lgr5^+^ hair follicle progenitors can be activated independent of Wnt ligands but *via* TNF produced by macrophages (42–46). Similarly, Lim et al. have shown that the interfollicular epidermal progenitors can self-renewal *via* autocrine Wnt signaling (39).

Sustained Wnt signaling in dermal fibroblasts may cause wound fibrosis whereases restraining Wnt activity using β-catenin inhibitors can reduce scarring (47–49). Wnt signaling also enhanced myofibroblast proliferation and expression of profibrotic genes, including TGF-β1 and extracellular protein coding gene (47, 48). However, our data show that collagen composition in the *Wls^fl/fl^LysM-Cre^+^* wound was not altered (**Figures 3C, D**), suggesting that myeloid-derived Wnt ligands contributed minimally to scar formation or fibrosis after small wound excision.

Wnt signaling controls monocyte cell fate determination and macrophage differentiation (50, 51). Flow cytometry analysis of the unwounded skin and wound granulation in our study did not show any difference in the overall percentage of different myeloid cell populations after *Wls* knockout, indicating that myeloid-derived Wnt production is not required for myeloid cell recruitment and maintenance (**Figures 2B, C**). Similar to our findings, several studies using either the *Wls^fl/fl^ Csfr1-icre^+^ or Wls^fl/fl^ LysM-Cre^+^* mice have demonstrated that macrophage *Wls* abrogation did not change the spatial distribution of macrophages in homeostasis or after injury (19, 20, 24, 51, 52). Overall, our data indicates that myeloid-derived Wnt ligands are dispensable for blood vessel formation during adult skin homeostasis and cutaneous wound healing. Nevertheless, myeloid-derived Wnts may be involved in regulating endovascular progenitors’ self-renewal during acute wound healing. Notwithstanding, the kinetics of EVPs and implication of Wnt signaling in chronic wound remains largely unexplored. As such, our research findings might not be applicable in a chronic wound scenario and the regulatory function of myeloid-derived Wnts in chronic wound will require further investigation. Overall, these findings highlight the possible challenges in targeting Wnt signaling during wound healing given the multiplicity of cell types producing or reacting to Wnt ligands.

## Data availability statement

The original contributions presented in the study are included in the article/**Supplementary Material**. Further inquiries can be directed to the corresponding author.

## Ethics statement

All animal procedures in this study adhered to the guidelines of the National Health and Medical Research Council Australian Code for the Care and Use of Animals for Scientific Purposes, and were approved by The University of Queensland (UQ) Health Sciences Ethics Committee.

## Author contributions

SLS performed the experiments. SLS, SK and KK designed the experiments, analyzed and interpreted data, prepared figures and manuscripts. SLS, AB, SK and KK provided intellectual input to the study. SLS, SK and KK wrote the manuscript with SK and KK contribute equally as senior authors and editorial input from AB. AB, SK and KK supervised the study. All authors read and approved the final manuscript.

## Funding

AB acknowledges funding support through a National Health and Medical Research Council of Australia Project Grant (GNT1142456) and The University of Queensland Diamantina Institute. SLS acknowledges support from the University of Queensland for Postgraduate Scholarship through the Australian Government Research Training Program (RTP).

## Acknowledgments

We would like to thank TRI and UQBR animal facility for their daily assistance in caring for the animals used in this study. We also acknowledge Translational Research Institute’s flow cytometry and histology facility for providing technical support. Special thanks to Miss Lynn Tolley for her technical assistance in managing and genotyping the animals.

## Conflict of interest

The authors declare that the research was conducted in the absence of any commercial or financial relationships that could be construed as a potential conflict of interest.

## Publisher’s note

All claims expressed in this article are solely those of the authors and do not necessarily represent those of their affiliated organizations, or those of the publisher, the editors and the reviewers. Any product that may be evaluated in this article, or claim that may be made by its manufacturer, is not guaranteed or endorsed by the publisher.

## References

[1] Lukowski SW Patel J Andersen SB Sim S-L Wong HY Tay J. Single-cell transcriptional profiling of aortic endothelium identifies a hierarchy from endovascular progenitors to differentiated cells. Cell Rep (2019) 27(9):2748–58.e3. doi: 10.1016/j.celrep.2019.04.102 31141696

[2] Patel J Seppanen EJ Rodero MP Wong HY Donovan P Neufeld Z. Functional definition of progenitors versus mature endothelial cells reveals key soxf-dependent differentiation process. Circulation (2017) 135(8):786–805. doi: 10.1161/CIRCULATIONAHA.116.024754 27899395

[3] Zhao J Patel J Kaur S Sim S-L Wong HY Styke C. Sox9 and rbpj differentially regulate endothelial to mesenchymal transition and wound scarring in murine endovascular progenitors. Nat Commun (2021) 12(1):2564. doi: 10.1038/s41467-021-22717-9 33963183 PMC8105340

[4] Donovan P Patel J Dight J Wong HY Sim S-L Murigneux V. Endovascular progenitors infiltrate melanomas and differentiate towards a variety of vascular beds promoting tumor metastasis. Nat Commun (2019) 10(1):18. doi: 10.1038/s41467-018-07961-w 30604758 PMC6318267

[5] Nusse R. Wnt signaling and stem cell control. Cell Res (2008) 18(5):523–7. doi: 10.1038/cr.2008.47 18392048

[6] Van Camp JK Beckers S Zegers D Van Hul W. Wnt signaling and the control of human stem cell fate. Stem Cell Rev Rep (2014) 10(2):207–29. doi: 10.1007/s12015-013-9486-8 24323281

[7] Daneman R Agalliu D Zhou L Kuhnert F Kuo CJ Barres BA. Wnt/β-catenin signaling is required for cns, but not non-cns, angiogenesis. Proc Natl Acad Sci (2009) 106(2):641. doi: 10.1073/pnas.0805165106 19129494 PMC2626756

[8] Kato M Patel MS Levasseur R Lobov I Chang BHJ Glass DA II. Cbfa1-independent decrease in osteoblast proliferation, osteopenia, and persistent embryonic eye vascularization in mice deficient in Lrp5, a wnt coreceptor. J Cell Biol (2002) 157(2):303–14. doi: 10.1083/jcb.200201089 PMC2199263 11956231

[9] Phng L-K Potente M Leslie JD Babbage J Nyqvist D Lobov I. Nrarp coordinates endothelial notch and wnt signaling to control vessel density in angiogenesis. Dev Cell (2009) 16(1):70–82. doi: 10.1016/j.devcel.2008.12.009 19154719 PMC8114544

[10] Wang Y Rattner A Zhou Y Williams J Smallwood Philip M Nathans J. Norrin/Frizzled4 signaling in retinal vascular development and blood brain barrier plasticity. Cell (2012) 151(6):1332–44. doi: 10.1016/j.cell.2012.10.042 PMC3535266 23217714

[11] Zhang C Lai MB Khandan L Lee LA Chen Z Junge HJ. Norrin-induced Frizzled4 endocytosis and endo-lysosomal trafficking control retinal angiogenesis and barrier function. Nat Commun (2017) 8(1):16050. doi: 10.1038/ncomms16050 28675177 PMC5500887

[12] Zhou Y Wang Y Tischfield M Williams J Smallwood PM Rattner A. Canonical wnt signaling components in vascular development and barrier formation. J Clin Invest (2014) 124(9):3825–46. doi: 10.1172/JCI76431 PMC4151216 25083995

[13] Korn C Scholz B Hu J Srivastava K Wojtarowicz J Arnsperger T. Endothelial cell-derived non-canonical wnt ligands control vascular pruning in angiogenesis. Development (2014) 141(8):1757–66. doi: 10.1242/dev.104422 24715464

[14] Abtin A Jain R Mitchell AJ Roediger B Brzoska AJ Tikoo S. Perivascular macrophages mediate neutrophil recruitment during bacterial skin infection. Nat Immunol (2014) 15(1):45–53. doi: 10.1038/ni.2769 24270515 PMC4097073

[15] Barreiro O Cibrian D Clemente C Alvarez D Moreno V Valiente Í. Pivotal role for skin transendothelial radio-resistant anti-inflammatory macrophages in tissue repair. eLife (2016) 5:e15251. doi: 10.7554/Elife.15251 27304075 PMC4961461

[16] Honda M Surewaard BGJ Watanabe M Hedrick CC Lee W-Y Brown K. Perivascular localization of macrophages in the intestinal mucosa is regulated by Nr4a1 and the microbiome. Nat Commun (2020) 11(1):1329. doi: 10.1038/s41467-020-15068-4 32165624 PMC7067862

[17] Malsin ES Kim S Lam AP Gottardi CJ. Macrophages as a source and recipient of wnt signals. Front Immunol (2019) 10:1813(1813). doi: 10.3389/fimmu.2019.01813 31417574 PMC6685136

[18] Lobov IB Rao S Carroll TJ Vallance JE Ito M Ondr JK. Wnt7b mediates macrophage-induced programmed cell death in patterning of the vasculature. Nature (2005) 437(7057):417–21. doi: 10.1038/nature03928 PMC4259146 16163358

[19] Muley A Odaka Y Lewkowich IP Vemaraju S Yamaguchi TP Shawber C. Myeloid wnt ligands are required for normal development of dermal lymphatic vasculature. PloS One (2017) 12(8):e0181549. doi: 10.1371/journal.pone.0181549 28846685 PMC5573294

[20] Irvine KM Clouston AD Gadd VL Miller GC Wong W-Y Melino M. Deletion of wntless in myeloid cells exacerbates liver fibrosis and the ductular reaction in chronic liver injury. Fibrogenesis Tissue Repair (2015) 8(1):19. doi: 10.1186/s13069-015-0036-7 26473015 PMC4606475

[21] Kling JC Jordan MA Pitt LA Meiners J Thanh-Tran T Tran LS. Temporal regulation of natural killer T cell interferon gamma responses by β-Catenin-Dependent and -independent wnt signaling. Front Immunol (2018) 9:483(483). doi: 10.3389/fimmu.2018.00483 29616022 PMC5864864

[22] Lee J Rodero MP Patel J Moi D Mazzieri R Khosrotehrani K. Interleukin-23 regulates interleukin-17 expression in wounds, and its inhibition accelerates diabetic wound healing through the alteration of macrophage polarization. FASEB J (2018) 32(4):2086–94. doi: 10.1096/fj.201700773R 29208701

[23] Toda G Yamauchi T Kadowaki T Ueki K. Preparation and culture of bone marrow-derived macrophages from mice for functional analysis. STAR Protoc (2021) 2(1):100246. doi: 10.1016/j.xpro.2020.100246 33458708 PMC7797923

[24] Jiang A Okabe H Popovic B Preziosi ME Pradhan-Sundd T Poddar M. Loss of wnt secretion by macrophages promotes hepatobiliary injury after administration of 3,5-Diethoxycarbonyl-1, 4-dihydrocollidine diet. Am J Pathol (2019) 189(3):590–603. doi: 10.1016/j.ajpath.2018.11.010 30610845 PMC6436111

[25] Maiti G Naskar D Sen M. The wingless homolog Wnt5a stimulates phagocytosis but not bacterial killing. Proc Natl Acad Sci (2012) 109(41):16600. doi: 10.1073/pnas.1207789109 23012420 PMC3478623

[26] Ljungberg JK Kling JC Tran TT Blumenthal A. Functions of the wnt signaling network in shaping host responses to infection. Front Immunol (2019) 10:2521. doi: 10.3389/fimmu.2019.02521 31781093 PMC6857519

[27] Rodrigues M Kosaric N Bonham CA Gurtner GC. Wound healing: A cellular perspective. Physiol Rev (2019) 99(1):665–706. doi: 10.1152/physrev.00067.2017 30475656 PMC6442927

[28] Rodero MP Khosrotehrani K. Skin wound healing modulation by macrophages. Int J Clin Exp Pathol (2010) 3(7):643–53. PMC2933384 20830235

[29] Rodero MP Legrand JMD Bou-Gharios G Khosrotehrani K. Wound-associated macrophages control collagen 1α2 transcription during the early stages of skin wound healing. Exp Dermatol (2013) 22(2):143–5. doi: 10.1111/exd.12068 23278967

[30] Chaudet KM Goyal A Veprauskas KR Nazarian RM. Wnt signaling pathway proteins in scar, hypertrophic scar, and keloid: Evidence for a continuum? Am J Dermatopathol (2020) 42(11):842–7. doi: 10.1097/DAD.0000000000001661 32310858

[31] Jridi I Canté-Barrett K Pike-Overzet K Staal FJT. Inflammation and wnt signaling: Target for immunomodulatory therapy? Front Cell Dev Biol (2021) 8:615131. doi: 10.3389/fcell.2020.615131 33614624 PMC7890028

[32] Chavali M Ulloa-Navas MJ Pérez-Borredá P Garcia-Verdugo JM McQuillen PS Huang EJ. Wnt-dependent oligodendroglial-endothelial interactions regulate white matter vascularization and attenuate injury. Neuron (2020) 108(6):1130–45.e5. doi: 10.1016/j.neuron.2020.09.033 33086038 PMC7769920

[33] Okuse T Chiba T Katsuumi I Imai K. Differential expression and localization of wnts in an animal model of skin wound healing. Wound Repair Regeneration (2005) 13(5):491–7. doi: 10.1111/j.1067-1927.2005.00069.x 16176457

[34] Stefater Iii JA Lewkowich I Rao S Mariggi G Carpenter AC Burr AR. Regulation of angiogenesis by a non-canonical wnt–Flt1 pathway in myeloid cells. Nature (2011) 474(7352):511–5. doi: 10.1038/nature10085 PMC3214992 21623369

[35] Saha S Aranda E Hayakawa Y Bhanja P Atay S Brodin NP. Macrophage-derived extracellular vesicle-packaged wnts rescue intestinal stem cells and enhance survival after radiation injury. Nat Commun (2016) 7(1):13096. doi: 10.1038/ncomms13096 27734833 PMC5065628

[36] Valenta T Degirmenci B Moor AE Herr P Zimmerli D Moor MB. Wnt ligands secreted by subepithelial mesenchymal cells are essential for the survival of intestinal stem cells and gut homeostasis. Cell Rep (2016) 15(5):911–8. doi: 10.1016/j.celrep.2016.03.088 27117411

[37] Boulter L Govaere O Bird TG Radulescu S Ramachandran P Pellicoro A. Macrophage-derived wnt opposes notch signaling to specify hepatic progenitor cell fate in chronic liver disease. Nat Med (2012) 18(4):572–9. doi: 10.1038/nm.2667 PMC3364717 22388089

[38] Choi Yeon S Zhang Y Xu M Yang Y Ito M Peng T. Distinct functions for Wnt/β-catenin in hair follicle stem cell proliferation and survival and interfollicular epidermal homeostasis. Cell Stem Cell (2013) 13(6):720–33. doi: 10.1016/j.stem.2013.10.003 PMC3900235 24315444

[39] Lim X Tan SH Koh WLC Chau RMW Yan KS Kuo CJ. Interfollicular epidermal stem cells self-renew *Via* autocrine wnt signaling. Science (2013) 342(6163):1226. doi: 10.1126/science.1239730 24311688 PMC4081860

[40] Scholz B Korn C Wojtarowicz J Mogler C Augustin I Boutros M. Endothelial Rspo3 controls vascular stability and pruning through non-canonical Wnt/Ca<Sup>2+</Sup>/Nfat signaling. Dev Cell (2016) 36(1):79–93. doi: 10.1016/j.devcel.2015.12.015 26766444

[41] Shi Y Shu B Yang R Xu Y Xing B Liu J. Wnt and notch signaling pathway involved in wound healing by targeting c-myc and Hes1 separately. Stem Cell Res Ther (2015) 6(1):120. doi: 10.1186/s13287-015-0103-4 26076648 PMC4501079

[42] Huang S Kuri P Aubert Y Brewster M Li N Farrelly O. Lgr6 marks epidermal stem cells with a nerve-dependent role in wound re-epithelialization. Cell Stem Cell (2021) 28(9):1582–96.e6. doi: 10.1016/j.stem.2021.05.007 34102139 PMC8528178

[43] Joost S Jacob T Sun X Annusver K La Manno G Sur I. Single-cell transcriptomics of traced epidermal and hair follicle stem cells reveals rapid adaptations during wound healing. Cell Rep (2018) 25(3):585–97.e7. doi: 10.1016/j.celrep.2018.09.059 30332640

[44] Aragona M Dekoninck S Rulands S Lenglez S Mascré G Simons BD. Defining stem cell dynamics and migration during wound healing in mouse skin epidermis. Nat Commun (2017) 8(1):14684. doi: 10.1038/ncomms14684 28248284 PMC5339881

[45] Ito M Liu Y Yang Z Nguyen J Liang F Morris RJ. Stem cells in the hair follicle bulge contribute to wound repair but not to homeostasis of the epidermis. Nat Med (2005) 11(12):1351–4. doi: 10.1038/nm1328 16288281

[46] Wang X Chen H Tian R Zhang Y Drutskaya MS Wang C. Macrophages induce Akt/β-Catenin-Dependent Lgr5+ stem cell activation and hair follicle regeneration through tnf. Nat Commun (2017) 8(1):14091. doi: 10.1038/ncomms14091 28345588 PMC5378973

[47] Gay D Ghinatti G Guerrero-Juarez CF Ferrer RA Ferri F Lim CH. Phagocytosis of wnt inhibitor Sfrp4 by late wound macrophages drives chronic wnt activity for fibrotic skin healing. Sci Adv (2020) 6(12):eaay3704. doi: 10.1126/sciadv.aay3704 32219160 PMC7083618

[48] Hamburg-Shields E DiNuoscio GJ Mullin NK Lafyatis R Atit RP. Sustained β-catenin activity in dermal fibroblasts promotes fibrosis by up-regulating expression of extracellular matrix protein-coding genes. J Pathol (2015) 235(5):686–97. doi: 10.1002/path.4481 PMC4357547 25385294

[49] Bastakoty D Saraswati S Cates J Lee E Nanney LB Young PP. Inhibition of Wnt/β-catenin pathway promotes regenerative repair of cutaneous and cartilage injury. FASEB J (2015) 29(12):4881–92. doi: 10.1096/fj.15-275941 PMC4653050 26268926

[50] Brown AL Salerno DG Sadras T Engler GA Kok CH Wilkinson CR. The gm-csf receptor utilizes β-catenin and Tcf4 to specify macrophage lineage differentiation. Differentiation (2012) 83(1):47–59. doi: 10.1016/j.diff.2011.08.003 22099176 PMC3394929

[51] Sessa R Yuen D Wan S Rosner M Padmanaban P Ge S. Monocyte-derived Wnt5a regulates inflammatory lymphangiogenesis. Cell Res (2016) 26(2):262–5. doi: 10.1038/cr.2015.105 PMC4746601 26337801

[52] Stefater JA III Rao S Bezold K Aplin AC Nicosia RF Pollard JW. Macrophage wnt-Calcineurin-Flt1 signaling regulates mouse wound angiogenesis and repair. Blood (2013) 121(13):2574–8. doi: 10.1182/blood-2012-06-434621 PMC3612865 23303818

