# Supplementary material for: Myeloid Wls expression is dispensable for skin wound healing and blood vessel regeneration
Source: Front Endocrinol (Lausanne). 2022 Aug 22;13:957833. doi: 10.3389/fendo.2022.957833 (PMC9446346; doi:10.3389/fendo.2022.957833)
Supplement: Supplementary file 1 [file DataSheet_1.pdf]

# *Supplementary Material*

**Supplementary Figure 1**

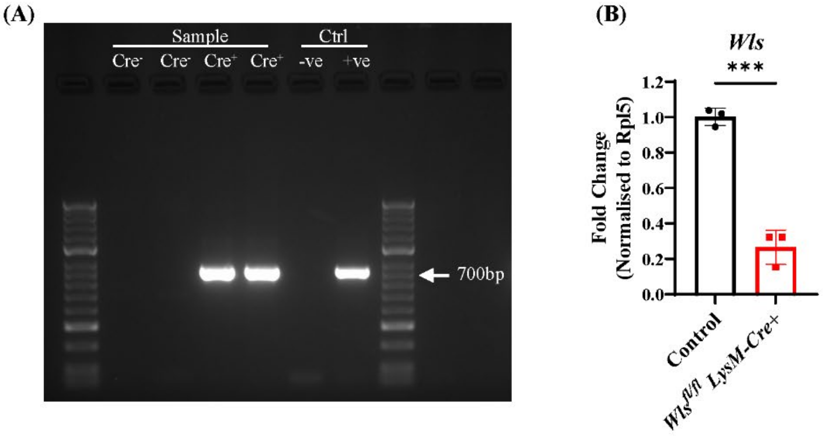


**Supplementary Figure 1: Bone marrow-derived macrophages from *Wls$^{fl/fl}$ LysM-Cre$^{+}$* mice express lower level of *Wls* mRNA. A)** Genotyping of the *Wls$^{fl/fl}$ LysM-Cre$^{+}$* animals for the Cre recombinase gene (700bp). **B)** Bone marrow-derived macrophages from the *Wls$^{fl/fl}$ LysM-Cre$^{+}$* mice expressed lower *Wls* mRNA compared to the *Wls$^{fl/fl}$ LysM-Cre$^{neg}$* control littermates. Data are represented as mean ± SD. Statistical analysis was performed using unpaired Student t-tests. *** = $p<0.0005$. Data are representative of 3 individual animal samples.

## Supplementary Figure 2

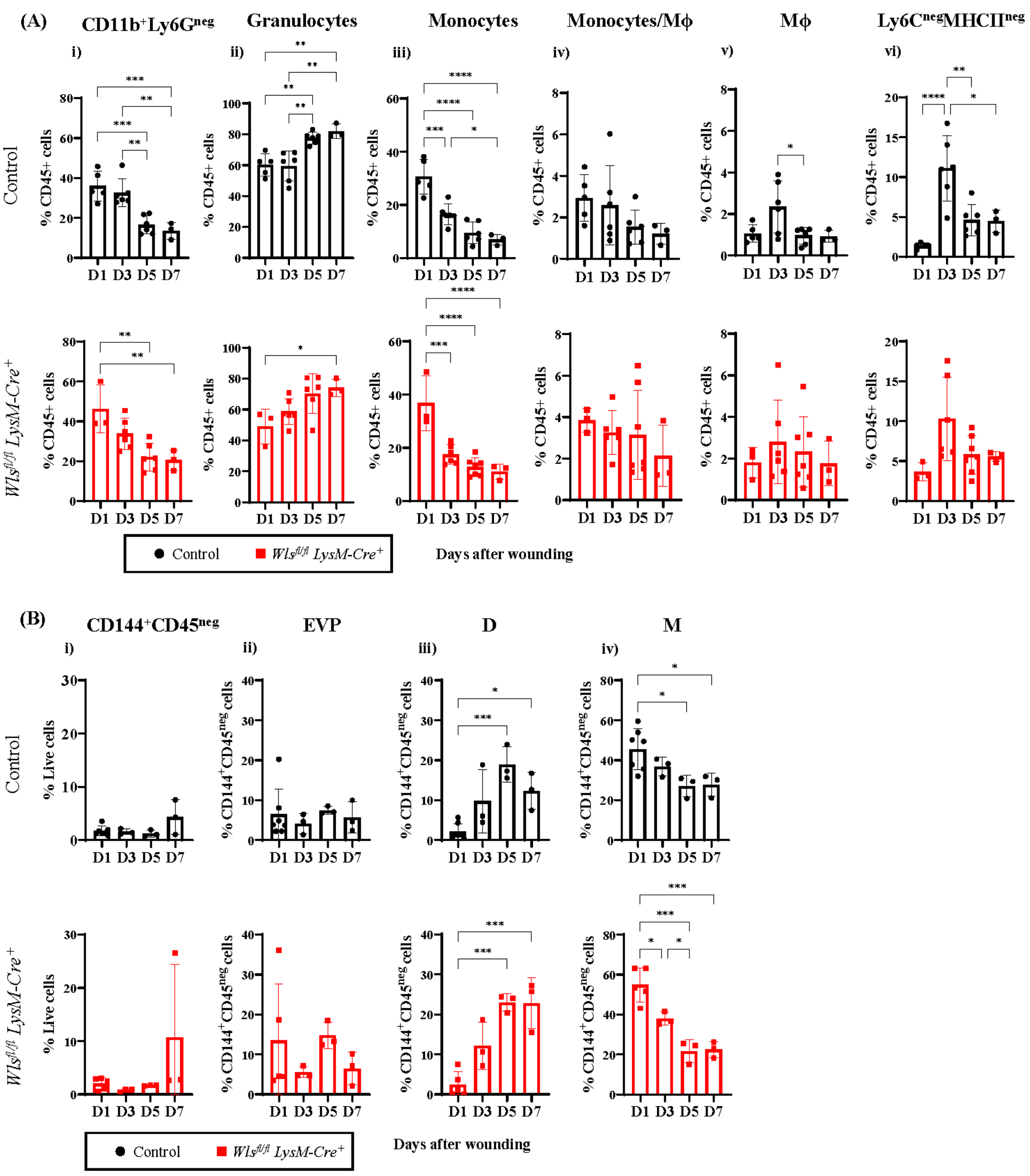


**Supplementary Figure 2: Kinetics of myeloid and endothelial cells infiltration into the granulation tissue during the seven days wound healing period. A)** Flow cytometry quantification of **(i)** CD11b$^{+}$Ly6G$^{neg}$, **(ii)** Granulocytes, **(iii)** Monocytes, **(iv)** Monocytes/Mϕ, **(v)** Mϕ, and **(vi)** Ly6C$^{neg}$MHCII$^{neg}$ between day 1, 3, 5 and 7. Highest percentage of monocytes were identified at day 1, followed by a gradual decline between day 3 and day 7. The percentage of macrophage and

$Ly6C^{neg}MHCII^{neg}$ cells peaked at day 3 and reduced at day 5 and day 7. **B)** Progressive changes in the **(i)** $CD144^{+}CD45^{neg}$, **(ii)** EVP, **(iii)** D, and **(iv)** M cells over time during the various wound healing phases. EVP remains consistent across the wound healing period in the *Wls*$^{fl/fl}$ *LysM-Cre*$^{neg}$ animals, and a notable increased of EVP in the *Wls*$^{fl/fl}$ *LysM-Cre*$^{+}$ at day 5. D cells increased significantly between day 3 and day 5 for both *Wls*$^{fl/fl}$ *LysM-Cre*$^{+}$ and control animals. Percentage of the M population declined across the seven days of wound healing. Data are represented as mean ± SD. Statistical analysis was performed using two-way ANOVA. * = p<0.05, ** = p<0.005 and *** = p<0.0005. Data are representative of 3-6 individual animal samples.

**Supplementary Figure 3**

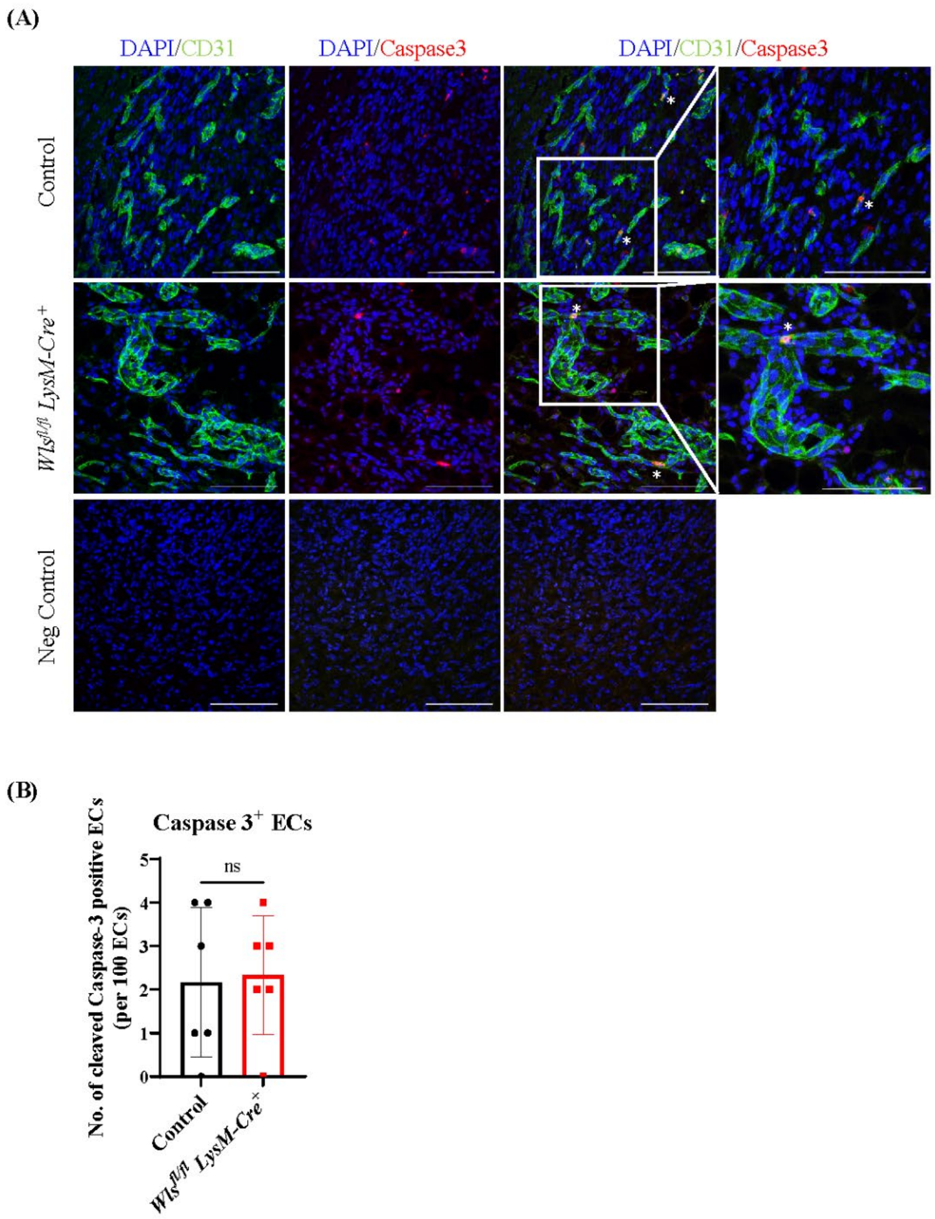


**Supplementary Figure 3: Myeloid *Wls* activity does not regulate endothelial cell apoptosis. A)** Double immunofluorescence staining of the apoptosis marker, cleaved Caspase-3 with CD31 in day 5 wounds (scale bar = 100µm). **B)** Quantification of the immunofluorescence staining showed similar number of Caspase-3$^{+}$ endothelial cells between the control and *Wls*$^{fl/fl}$ *LysM-Cre*$^{+}$ mice. Data are represented as mean ± SD. Statistical analysis was performed using unpaired Student t-tests. ns = not significant. ECs = endothelial cells. Data are representative of 6 individual animal samples.
